# Kaposiform hemangioendothelioma: current knowledge and future perspectives

**DOI:** 10.1186/s13023-020-1320-1

**Published:** 2020-02-03

**Authors:** Yi Ji, Siyuan Chen, Kaiying Yang, Chunchao Xia, Li Li

**Affiliations:** 1 Division of Oncology, Department of Pediatric Surgery, West China Hospital of Sichuan University, Chengdu, 610041 China; 2 Pediatric Intensive Care Unit, Department of Critical Care Medicine, West China Hospital of Sichuan University, #37 Guo-Xue-Xiang, Chengdu, 610041 China; 3 Department of Radiology, West China Hospital of Sichuan University, Chengdu, 610041 China; 4 Laboratory of Pathology, West China Hospital of Sichuan University, Chengdu, 610041 China

**Keywords:** Kaposiform hemangioendothelioma, Kasabach-Merritt phenomenon, Angiogenesis, Lymphangiogenesis, Treatment

## Abstract

Kaposiform hemangioendothelioma (KHE) is a rare vascular neoplasm with high morbidity and mortality. The initiating mechanism during the pathogenesis of KHE has yet to be discovered. The main pathological features of KHE are abnormal angiogenesis and lymphangiogenesis. KHEs are clinically heterogeneous and may develop into a life-threatening thrombocytopenia and consumptive coagulopathy, known as the Kasabach-Merritt phenomenon (KMP). The heterogeneity and the highly frequent occurrence of disease-related comorbidities make the management of KHE challenging. Currently, there are no medications approved by the FDA for the treatment of KHE. Multiple treatment regimens have been used with varying success, and new clinical trials are in progress. In severe patients, multiple agents with variable adjuvant therapies are given in sequence or in combination. Recent studies have demonstrated a satisfactory efficacy of sirolimus, an inhibitor of mammalian target of rapamycin, in the treatment of KHE. Novel targeted treatments based on a better understanding of the pathogenesis of KHE are needed to maximize patient outcomes and quality of life. This review summarizes the epidemiology, etiology, pathophysiology, clinical features, diagnosis and treatments of KHE. Recent new concepts and future perspectives for KHE will also be discussed.

## Introduction

Kaposiform hemangioendothelioma (KHE) is a rare vascular neoplasm that is typically diagnosed in infancy or early childhood. KHE has intermediate tumor type with locally aggressive characteristics. The predominant feature of the pathology of KHE is progressive angiogenesis and lymphangiogenesis [1, 2]. Clinically, KHE has high morbidity rates, primarily due to local invasive features, compressive effects, or the life-threatening consumptive coagulopathy known as the Kasabach-Merritt phenomenon (KMP) [3–5]. Recent studies have rapidly expanded our basic knowledge of KHE, including the etiology, pathophysiology, diagnosis and treatment of the disorder. In this review, we describe the current knowledge and discuss future perspectives on KHE, with the aim of improving our understanding of KHE and preventing mortality and morbidity in severe cases.

## Definition

**Fig. 1:**
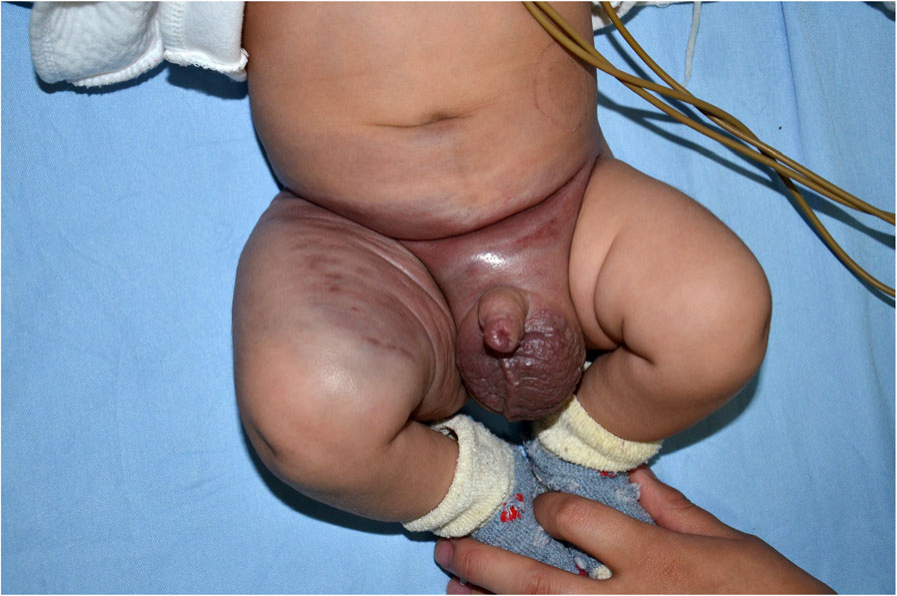
A 3-month-old male infant presenting KHE associated with KMP. The tumor lesion grew progressively after the first week of age and extended through the whole thigh, scrotum and abdominal wall, with ‘extensive thrombocytopenic purpura’ as described by Kasabach and Merritt in 1940

Since 1940, the term ‘Kasabach-Merritt syndrome (KMS)’ has been used for patients with vascular anomalies that are associated with thrombocytopenia and coagulopathy [6]. KMS has also been widely considered a complication of ‘hemangioma’. KHE was first designated by Zukerberg and coworkers in 1993 as an entity distinct from infantile hemangioma (IH) because of its locally invasive growth, aggressive course and ‘focal Kaposi-like appearance’ [7]. In 1997, investigators from two different groups reported that the vascular lesions associated with KMS (or KMP) were not IHs as was previously believed [8, 9]. Currently, KMP is defined as profound thrombocytopenia, together with consumptive coagulopathy and hypofibrinogenemia associated only with the vascular tumors, KHE and tufted angioma (TA) (Fig. 1) [10, 11]. Conceptually, KHE and TA are part of the same neoplastic spectrum and can be present in the same biopsy specimen of the same patient [12].

## Epidemiology and demography

Currently, there is a paucity of literature specifically addressing the incidence of KHE. In Massachusetts, the annual prevalence and incidence have been estimated at 0.91 and 0.071 per 100,000 children, respectively [13]. However, asymptomatic KHE lesions are less likely to be reported or diagnosed by pathology. Indeed, small asymptomatic or atypical KHEs can be misdiagnosed as unusual variants of IH or other vascular anomalies [14]. Therefore, the actual prevalence and incidence of KHE are most likely higher than those indicated in the limited published reports.

Previously, KHE was shown to have an equal sex predilection. However, a slight male predominance has been indicated by two large retrospective studies, both of which collected data from more than 100 patients with KHE [10, 11]. The distribution of age at onset has one peak within the first year of life when approximately 90% of KHE are evident. Approximately 50% of cutaneous lesions are visible or detectable at birth [14].

## Etiology

In general, the etiology of KHE remains largely unknown. Almost all cases of KHE arise without any obvious cause. In rare scenarios, signs and symptoms of KHE/TA can worsen with trauma or infections. There is also evidence that aggravation or manipulation of the tumor either from surgery or trauma can incite KMP, whether the patient had a history of KMP or not [15–17]. In addition, rapid enlargement of the lesion with severe KMP development shortly after vaccination has been reported in several patients [18–21]. These phenomena raise the intriguing possibility that physical trauma and the inflammatory response might contribute to the aggravation of KHE.

It is likely that the origin of KHE is multifactorial, with genetic factors being part of the contributing triggers, although the mutations in KHE tumor are sporadic rather than germline. Rapid advances in the area of molecular genetics have enabled the identification of somatic mutations in many types of vascular anomalies. Zhou and coworkers provided evidence of a somatic translocation between chromosomes 13 and 16 at the bands of 13q14 and 16p13.3 in 10% of metaphase cells in KHE lesions with the presence of normal cells in the karyotype [22]. Remarkably, the somatic activating *GNA14* c.614A > T (p.Gln205Leu) mutation was found in 1/3 of KHE specimens and in 1/4 of TA specimens, although these studies were weakened by small sample size [23]. Somatic mutations in *GNAQ* and its paralogues (e.g., *GNA11* and *GNA14*) have also been identified in many other vascular tumors [24, 25], vascular malformations [26, 27] and solid tumors [28, 29].

**Fig. 2:**
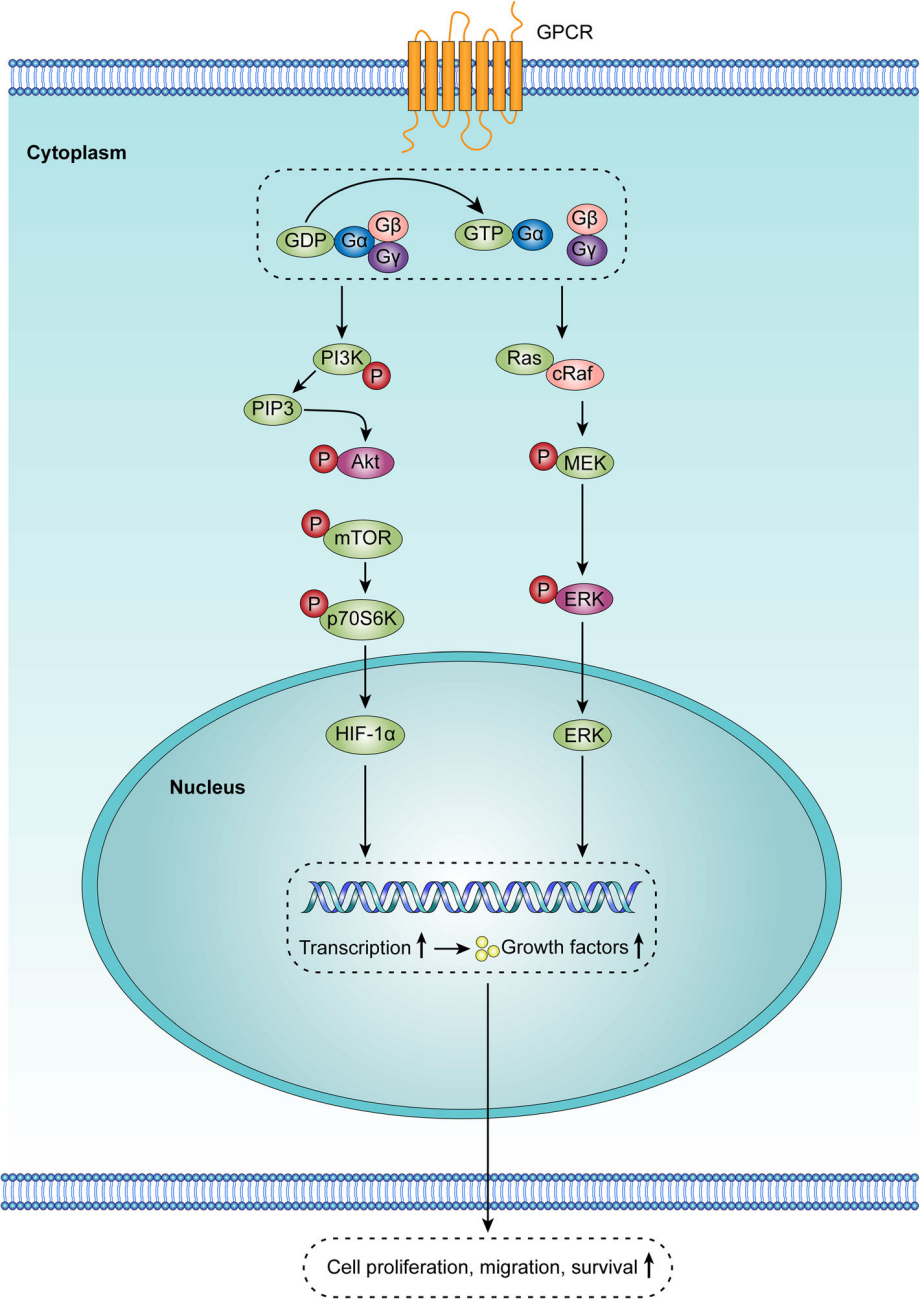
G-protein-coupled receptors (GPCRs) participate in different physiological processes. The binding of ligands to GPCRs triggers a universal G protein allosteric mechanism that promotes the exchange of GDP with GTP on the α subunit of heterotrimeric G proteins. This event causes the dissociation of Gα from the dimer. Gα subunits mediate signals between GPCRs and intracellular signaling cascades. These signaling pathways include the PI3K/AKT/mTOR and MAPK/ERK pathways, both of which can mediate different biological processes, such as cell proliferation, migration and survival. Mutations in GPCRs and G proteins have been found at a very high frequency in tumor cells and endothelial cells in vascular anomalies

The *GNAQ* family encodes Gα subunits that form a heterotrimer with Gβ and Gγ subunits and bind G-protein-coupled receptors (GPCRs). GPCRs are involved in many aspects of tumor and vascular biology [30, 31]. In addition, platelet aggregation, glucose secretion, and inflammation are among the physiological processes affected by GPCRs [32]. p.Gln205Leu substitution can induce changes in cellular morphology and render cells growth-factor independent by upregulating the MAPK/ERK1/2 pathway (Fig. 2) [23]. However, it is important to note that although GNAQ mutations have been found in KHE, we do not know whether they are causative or develop secondarily in the tumor.

It is unclear how mutations in the same gene can lead to different vascular anomalies or clinical manifestations, but the mechanism may be based on the location of the mutation in the gene, the cell type(s) affected, and/or the point in development at which the mutations occur [33]. In these scenarios, the highly variable clinical presentations of KHE further reflect the complexity of gene mutations in the development of this rare disease. It is also conceivable that undetected mutations exist in KHE lesions given that many technical hurdles are still present, although these are not likely to be insurmountable in the future.

## Pathophysiology

The pathophysiology of KHE may not be attributable to a single mechanism, but rather, to a combination of events that have not yet been elucidated or understood completely.

### Angiogenesis and lymphangiogenesis

KHE is the result of dysregulation of both angiogenesis and lymphangiogenesis. In vivo, mouse hemangioendothelioma cells can form KHE-like, intradermal tumors. Interestingly, overexpression of prospero-related homeobox-1 (Prox-1) in mouse hemangioendothelioma cells induces an invasive phenotype in vivo, enhances the migration rate in vitro, and significantly upregulates the expression of podoplanin (D2–40) and vascular endothelial growth factor receptor-3 (VEGFR-3) [34]. Recent data indicated that KHE-derived mesenchymal stem cells (MSCs) have the capacity to support vascular network formation in vitro [35]. In addition to expressing VEGFR-3, KHE-derived MSCs also show higher levels of VEGF-C than normal lymphatic endothelial cells [35].

### VEGF-C/VEGFR3 axis

**Fig. 3:**
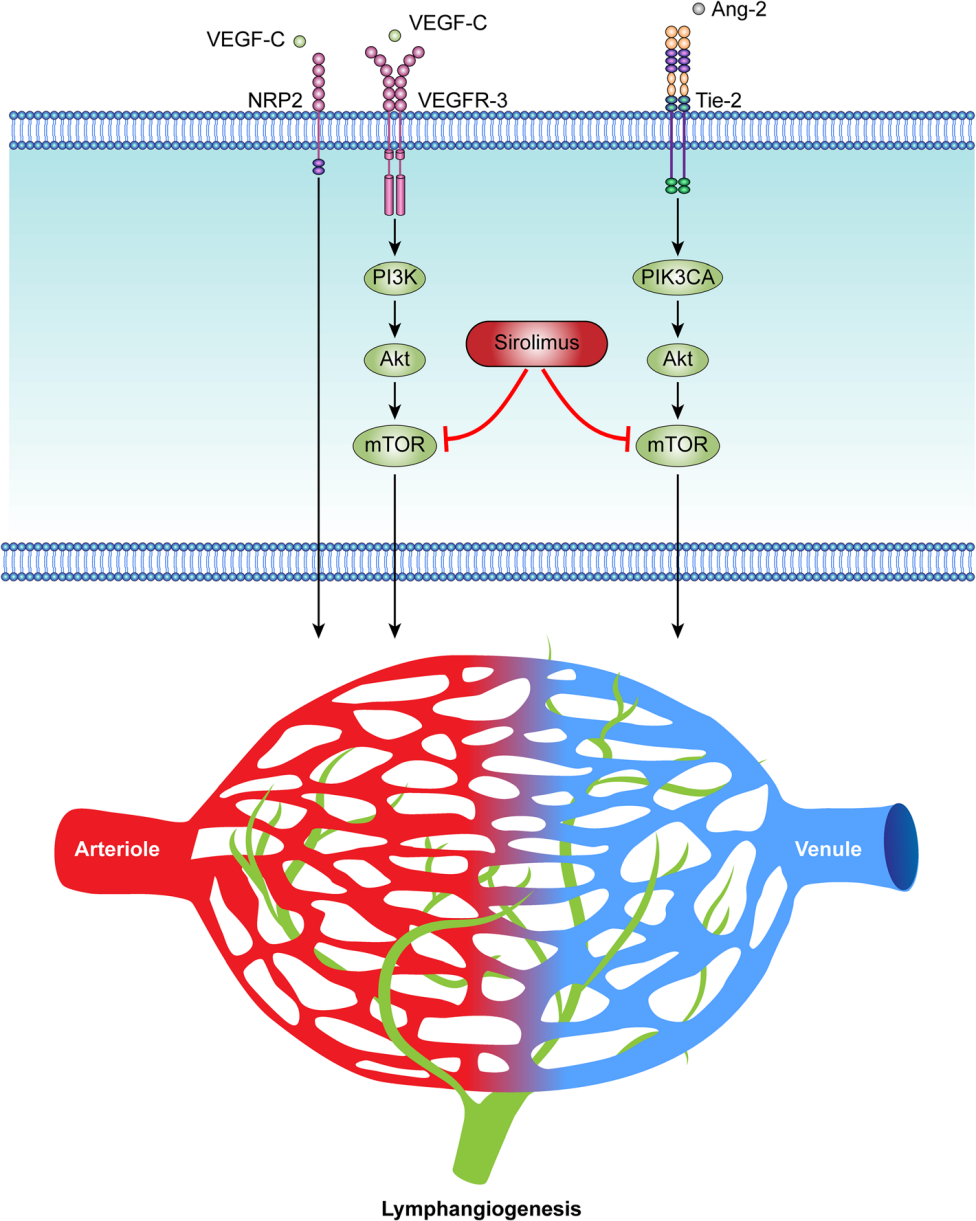
VEGF-C/VEGFR3 and Ang-2/Tie-2 signaling pathways play an important role in lymphangiogenesis. The binding of VEGF-C can stimulate the activation of VEGFR-3 and induce downstream PI3K/Akt/mTOR signaling, which mediates lymphangiogenesis. VEGF-C binding to NRP-2 can form a complex with VEGFR-3, further activating the VEGFR-3 signaling that enhances the proliferation of lymphatic endothelial cells (LECs) and lymphangiogenesis. Ang-2 ligand-induced Tie-2 activation triggers Akt/mTOR signaling, which in LECs is mainly mediated by PIK3CA. Gene-targeting experiments have identified that the Ang-2/Tie-2 signaling system is needed for physiological and pathological remodeling of lymphatic vessels. Sirolimus (rapamycin), which is an inhibitor of mTOR, complements current antilymphatic strategies in the treatment of vascular anomalies, such as KHE

The vascular endothelial growth factor-C (VEGF-C)/VEGFR3 axis in lymphatic endothelial cells (LECs) is important throughout lymphangiogenenic growth [36]. The expression of both VEGFR-3 and VEGF-C in KHE suggests that the VEGF-C/VEGFR3 axis may contribute to the aggressive behavior of KHE [37, 38]. The VEGF-C/VEGFR3 axis has been implicated in tumor progression by directly affecting tumor cells or modulating lymphangiogenesis and the immune response (Fig. 3) [39]. The VEGF-C/VEGFR-3 axis has been demonstrated to promote tumor growth in an autocrine manner [40]. In addition to lymphangiogenesis, VEGF-C/VEGFR3 signaling has also been shown to be important for angiogenesis, acting together with VEGF-A/VEGFR-2 and Dll4/Notch signaling to control angiogenesis [41]. VEGF-C/VEGFR3 axis may play an important role in chronic inflammation associated with KHE [42, 43].

### Angiopoietin-2/tie-2 signaling

The high serum levels of angiopoietin-2 (Ang-2) in patients with KHE raise the possibility that Ang-2 might have a paracrine effect on endothelial cells (ECs) in KHE lesions. Remarkably, Ang-2 levels dramatically decrease with sirolimus treatment in patients with KHE and KMP [44]. It is therefore possible that Ang-2 might have a role in the development of KMP in patients with KHE. The Ang/Tie2 ligand-receptor system is required for lymphatic and blood vessel development during midgestation. The Ang/Tie2 pathway controls vascular permeability, inflammation and pathological angiogenic and lymphatic responses postnatally. Ang-2 acts as a context-dependent weak Tie-2 agonist or antagonist that can inhibit the Ang-1/Tie-2 signaling axis [45]. In humans, Ang-2 levels are greatly increased during vascular remodeling, which occurs, for example, during vessel sprouting and inflammatory lymphangiogenesis [46]. This may be a potential explanation for the upregulation of Ang-2 in patients with KHE. Ang-2-induced Tie-2 activation triggers Akt/mTOR signaling, which in ECs is mainly mediated by PIK3CA (which encodes PI3K catalytic subunit-α) [47].

### Platelet aggregation: a key trigger of KMP?

Intralesional platelet trapping is followed by the activation and aggregation of platelets, which then results in activation of the coagulation cascade with subsequent consumption of clotting factors. Platelet trapping has been revealed histologically in KHE with or without KMP [48]. Of interest then is the mechanism or mechanisms of increased platelet trapping in KHE vessels. One hypothesis is that EC damage or alteration in KHE can lead to exposure of extracellular matrix components, which are ligands for platelet adhesion [1]. The C-type lectin-like receptor-2 (CLEC-2) expressed on platelets is an endogenous receptor of podoplanin, which in turn is widely expressed in ECs within the KHE lesions [49, 50]. Binding of podoplanin to CLEC-2 can transmit platelet activation signals via Src family kinases, which may account for platelet aggregation in KHE [51].

Podoplanin is highly expressed in dysmorphic vessels within the lymphatic malformations, but no obvious platelet aggregation occurs in these lesions [52]. Therefore, there may also be alternative and/or additional mechanisms by which platelet trapping is triggered in KHE lesions [2, 53]. In KHE, the thrombi in the microvasculature cause vessel occlusion and prevent normal blood flow, all of which can lead to elevated shear stress. It is recognized that high shear stress induces an increased activation of platelets in vitro and in vivo, with a mechanism dependent on von Willebrand factor interacting with both its platelet binding sites, glycoprotein (GP) Ib-IX and GP IIb-IIIa [54, 55]. With regard to KHE, platelets in circulating blood may become exposed to turbulent blood flow and high shear stress that results from the architecture of the small, convoluted and thrombus-obstructed vessels within the KHE lesions. This process in turn causes further platelet trapping and activation during the active phase of KHE.

Continued platelet aggregation, together with coagulopathy and hypofibrinogenemia with elevated D-dimer (coagulation markers), eventually result in intralesional hemorrhage, which clinically manifests as very purpuric, warm, painful, and rapidly enlarged tumor lesion [3]. In patients with KHE, the coexistence of KMP always represents aggressive tumor progression. Although the pathophysiological roles of activated platelets in the KHE tissue environment are not yet fully understood, these observations raise the intriguing possibility that the activated platelets might contribute to worsening coagulopathy by promoting critical processes such as neovascularization. This hypothesis is supported by findings that platelets are activated in different tumor vasculatures [56–59]. Platelets are reservoirs of proangiogenic proteins that are mainly stored in α-granules and secreted upon physiological and pathological stimulations. Various types of tumor cells and tumor-derived ECs can activate platelets by different mechanisms. There is plenty of evidence that activated platelets exert their pleiotropic effects on many biological processes central to angiogenesis, progression, inflammation and metastasis in various tumor types [57, 60, 61]. Improving our understanding of such platelet involvement in neovascularization may potentially help in the development of alternative treatment strategies for patients with KHE.

## Clinical characteristics

The manifestations of KHE are variable and range from cutaneous lesions with wide varieties of appearances to deep masses without cutaneous signs. The clinical features also differ substantially between patients with KMP and patients without KMP [13, 14, 48]. In the majority of patients, KHE is a single soft tissue mass with cutaneous findings that range from an erythematous papule, plaque, or nodule to an indurated, purple and firm tumor. With KMP, these lesions are purpuric, hot to the touch, swollen and very painful. Most patients experience progressive lesion enlargement and/or symptom progression [62–64]. However, a small but significant minority of KHEs do not grow [13, 48]. Approximately 12% of patients lack cutaneous involvement [14].

## Complications

Complications in patients with KHE are common. The complication severity strongly depends on the age, lesion size, lesion location, lesion extension into the deep tissue and vital organs, and associated hematologic abnormalities. It is prudent for clinicians to remain vigilant of potential complications and of risk factors that may herald future complications.

### Kasabach-Merritt phenomenon

KMP occurs with an estimated incidence of 42 to 71% [2, 13, 14, 64]. The thrombocytopenia is usually severe, with a median platelet count of 21 × 10^9^/L at the initial presentation of KMP [14]. KHE lesions with KMP have progressive engorgement and purpura. KMP can lead to significant pain and secondary bleeding. As far as we know, KHE appear to be congenital as the majority of cases are diagnosed in the newborn/infancy period. It is now thought that the few cases in which adults were found to have KHE or to develop KMP, occurred because of an inciting event like trauma or pregnancy. The risk of KMP is highest for congenital KHEs with a large size (especially > 8 cm in diameter) [48, 65]. Anatomic location may also be a predictor of KMP. Clinically, intrathoracic KHEs are frequently associated with KMP [66–68]. The frequency of KMP in the retroperitoneal KHEs was also high [13, 14]. Evidently, intrathoracic and retroperitoneal lesions tend to be more expansive and infiltrative and are more likely to develop KMP. The identification of the most dangerous factors associated with KMP will be enormously helpful to treating clinicians.

### Musculoskeletal disorders

**Fig. 4:**
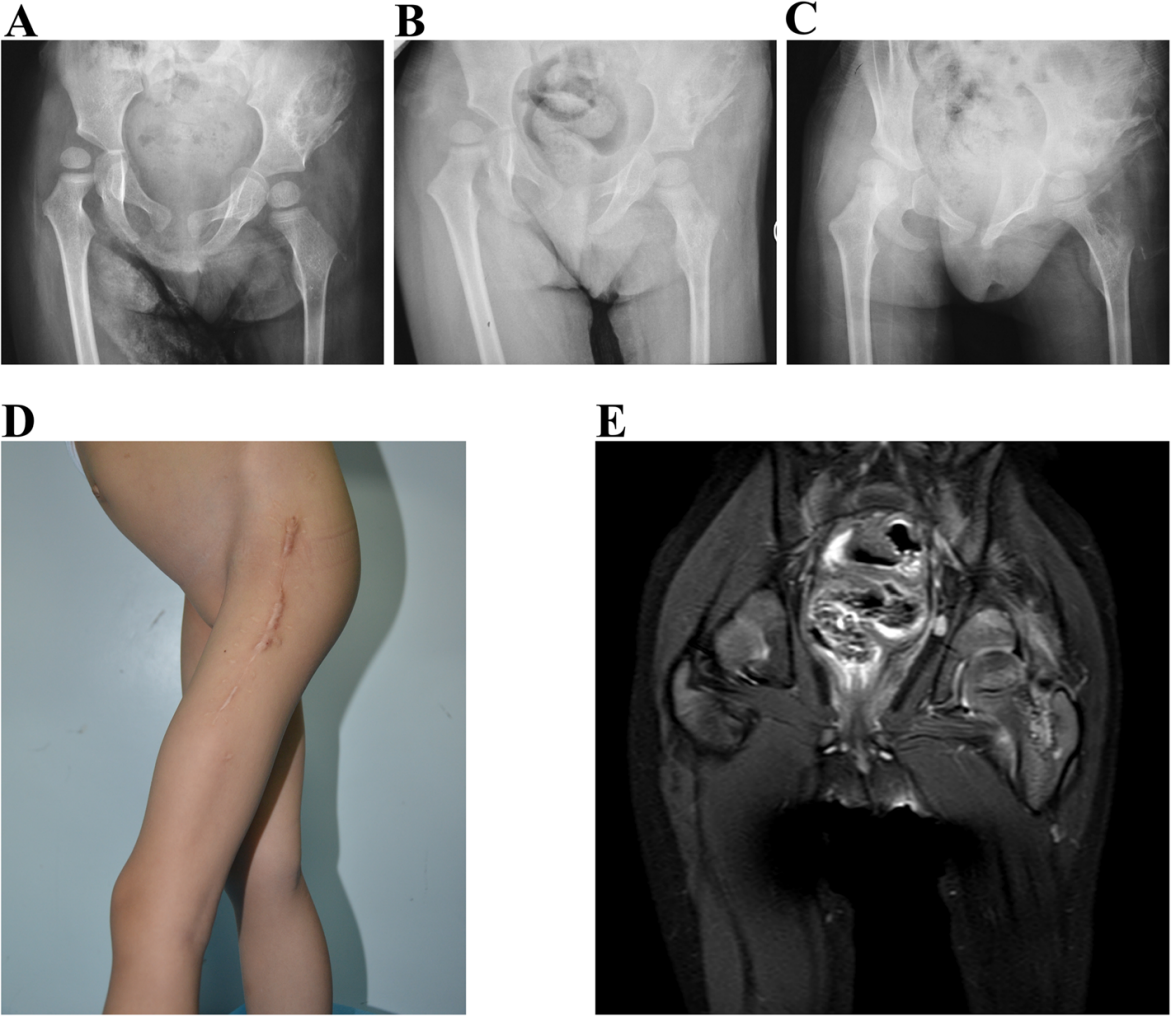
Deep KHE with bone and joint destruction in a 3.5-year-old girl. The patient had been diagnosed with a left-hip KHE associated with KMP at 1 year of age. She received a partial resection at the local hospital. Although surgical excision improved the associated KMP, she exhibited a progressively decreased hip range of motion. An anteroposterior pelvis radiograph showed right hip subluxation and progressive bone erosion in the left ilium and proximal femur before referral (**a**, **b** and **c**). Coronal T2-weighted MRI revealed a deep lesion infiltrating the left ilium and proximal femur at the time of referral to our department (**d**, **e**)

The infiltrative nature and destructive growth patterns of KHE can cause functional limitations and pain; all of these musculoskeletal disorders may affect patients’ abilities to perform routine daily activities and eventually influence quality of life [4, 69]. Acute pain at the tumor sites is a common symptom during KMP [14]. Even in patients without KMP, musculoskeletal disorders are frequently seen in cases involving the extremities, with a majority of these lesions located on or adjacent to joints [70]. Progressive thoracolumbar scoliosis can be seen in patients with thoracic or retroperitoneal lesions [71–73]. It is important that these patients are diagnosed early and treated more aggressively at the outset even in the absence of KMP. In some cases, residual KHEs will continue to infiltrate surrounding tissues, erode bone and destroy joints. Infiltration of the muscles and connective tissues in patients with KHE can alter the structural matrix and mechanical properties of these tissues, leading to chronic degenerative changes. Pathologically, untreated KHE lesions are characterized by progressive fibrosis [12]. There is ample evidence for the crucial role of platelet activation and aggregation in the development of fibrosis in different tissues and organs [74, 75]. It is conceivable that platelet activation and aggregation in muscles and connective tissues during KHE infiltration can activate variable fibrotic pathways. The diffuse intraarticular and periarticular fibrosis can further aggravate muscular atrophy and lead to subluxations and flexion contracture of the involved joints. Patients may eventually suffer from recalcitrant pain and fixed contractures (Fig. 4).

### Lymphedema

Lymphedema is the chronic, progressive swelling of subcutaneous tissue caused by inadequate lymphatic function. Lymphedema may be caused by anomalous development (primary) or injury (e.g., infection) to lymph nodes or lymphatic vessels [76]. Lymphedema may be a potential sequela of KHE, particularly tumor involving the legs [12, 77]. KHE involving the proximal extremity, particularly at or adjacent to the inguinal or axillary lymph nodes, may influence lymphatic development or damage the lymphatic vasculatures. In this scenario, it is hypothesized that the mechanical obstruction of the lymphatic flow during the acute phase of KMP may eventually lead to lymphedema [78]. In addition, active and chronic inflammation may also play a role in the development of lymphedema in patients with KHE.

### Compression of vital structures

Compression of vital structures may occur in a number of settings and is far more frequently observed in patients with KMP than in patients without KMP [14]. The progressive expansion of the mass during the active phase of KMP can further compromise the vital structures. Obstructing KHEs of the airway typically involves the neck and thorax [67, 79]. KHEs involving the pancreas are extremely uncommon but have the potential to cause obstructive jaundice [80, 81]. If compression of vital structures is visualized, prompt therapy should be initiated even without KMP.

## Diagnosis

The diagnosis of KHE often requires an analysis of clinical, imaging, hematological and/or histological characteristics, but even with all of these data, the diagnosis might not be readily apparent in certain cases [73]. In patients with deep KHE without KMP (bone and/or joint and so on), a definitive diagnosis is often delayed because of the non-specific and wide variety of symptoms.

**Fig. 5:**
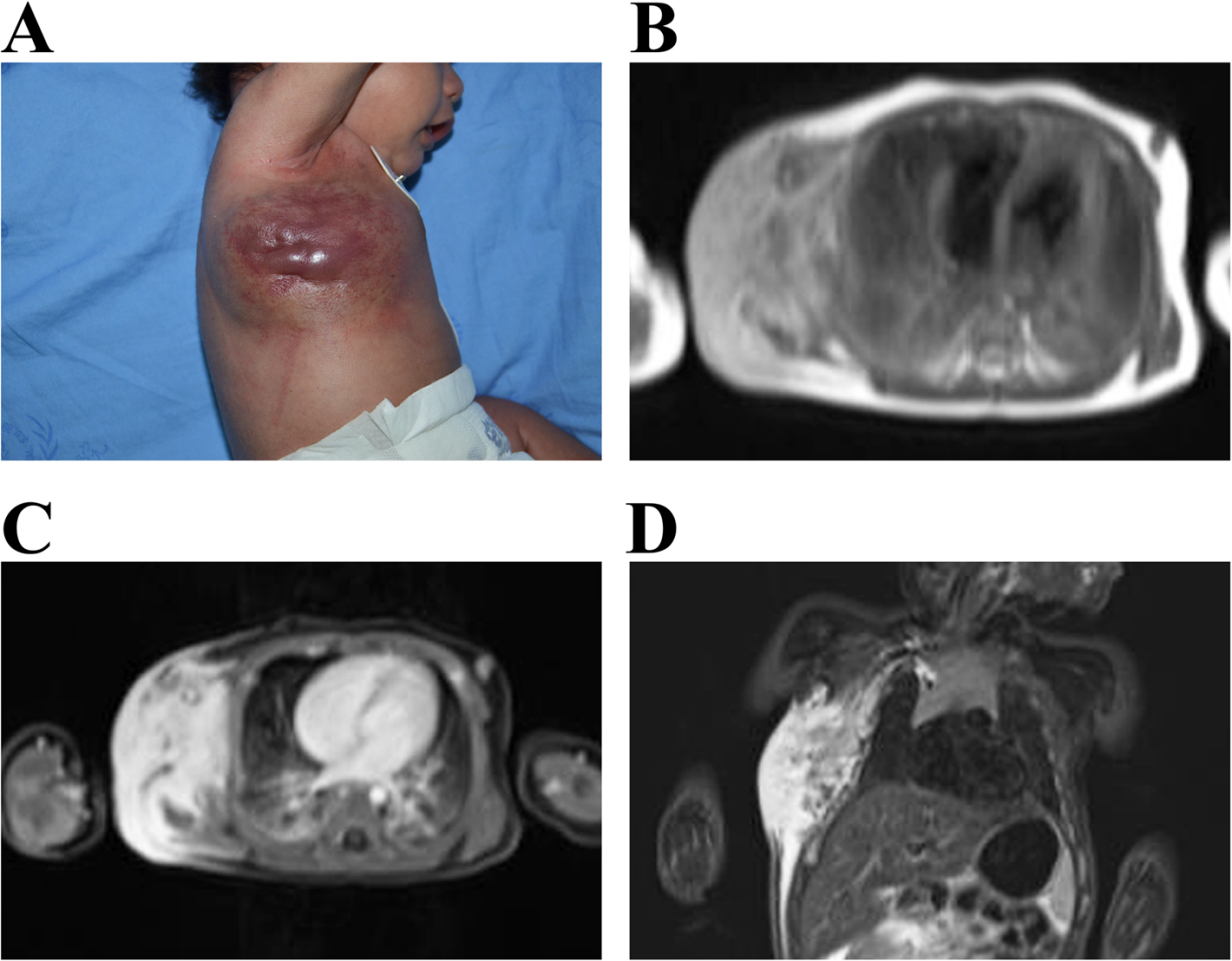
Clinical and MRI features of KHE with KMP. **a** A 2-month-old boy was found to have a chest wall mass after birth. The mass became progressively indurated and purpuric. The boy developed profound thrombocytopenia and consumptive coagulopathy. **b** Horizontal T1-weighted MRI revealed that the heterogeneous mass was isointense relative to the adjacent muscle on T1-weighted imaging. Horizontal (**c**) and sagittal (**d**) T2-weighted MRI revealed hyperintense lesions infiltrating the right lateral chest wall

Ultrasound is the modality of choice for small and superficial lesions [82]. Magnetic resonance imaging (MRI) is generally first-line assessment because the deep infiltrating nature of KHE may not be apparent on physical exam or on ultrasound. MRI with and without gadolinium has the most value in the diagnosis of KHE as well as for clearly determining the extent of involvement and response to treatment [83]. On MRI scans, KHEs usually exhibit ill-defined margins and are characterized by multiplanar involvement, diffuse enhancement, and adjacent fat stranding in unusual locations, iso-intensity relative to adjacent muscle on T1-weighted imaging, hyperintensity on T2-weighted imaging (Fig. 5). Adjacent bone and/or joint changes are common. The MRI findings of these changes include destruction of the adjacent cortex, injury of the epiphyseal region and invasion to near joints. Deep KHE with KMP should be considered in patients presenting with an unexplained severe thrombocytopenia and coagulopathy, especially in patients coexisting with cutaneous purpura and severe anemia. MRI scan of the abdomen and chest should be recommended for such patients [84, 85].

**Fig. 6:**
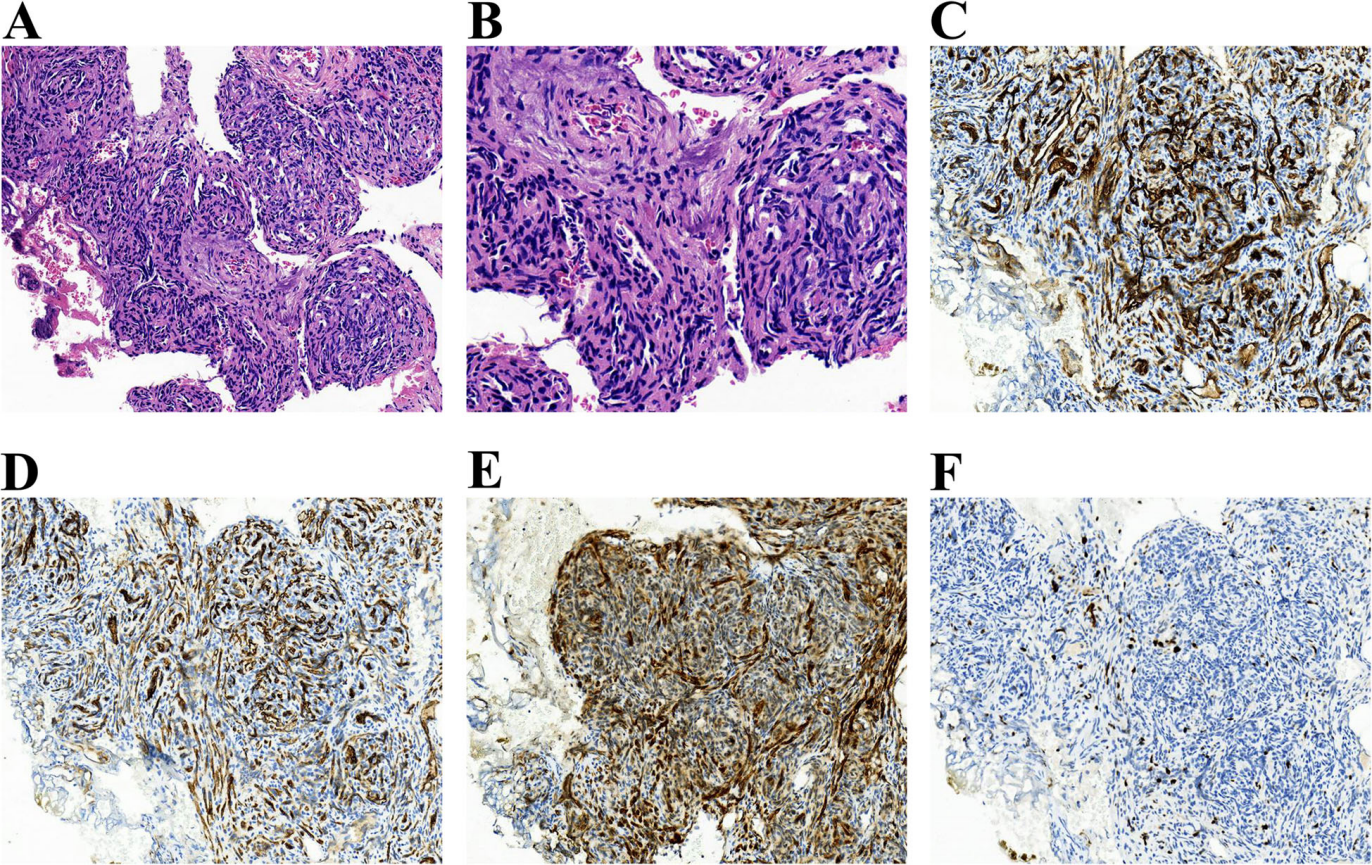
Histopathological characteristics of KHE. **a**Hematoxylin and eosin (H&E)-stained sections of KHE (original magnification × 100). The histologic hallmark of KHE was infiltrating, defined, rounded and confluent nodules, which were composed of spindle endothelial cells. **b** These spindle endothelial cells aligned to form malformed lymphatic channels and a slit-like vascular lumina (× 200). Immunohistochemical staining showed that the endothelial cells in KHE lesions were positive for the vascular endothelial markers CD31 (**c**, × 100) and CD34 (**d**, × 100) and the lymphatic endothelial marker D2–40 (**e**, × 100) but were negative for glucose transporter-1 and human herpes virus-8 staining (data not shown). Ki-67 was noted in only a few nuclei in the lesion (**f**, × 100)

Biopsy is gold standard for diagnosis and should be performed if possible and safe. Biopsy is frequently not possible or recommended in KHE with severe KMP, and can potentially worsen the coagulopathy. Biopsy specimens should be considered in patients with an uncertain diagnosis and atypical clinical manifestation (e.g., at an unusual site). The histologic hallmark of KHE is infiltrating, defined, rounded and confluent nodules, which are composed of spindle endothelial cells. These spindle endothelial cells align to form malformed lymphatic channels and slit-like vascular lumina containing erythrocytes, along with platelet thrombi, eosinophilic hyaline bodies and the extravasation of hemosiderin deposits. Immunohistochemical staining shows that endothelial cells in KHE lesions are positive both for vascular endothelial markers CD31 and CD34, lymphatic endothelial marker VEGFR-3, D2–40, lymphatic endothelial hyaluronan receptor-1 and Prox-1, but negative for glucose transporter-1(Glut-1) and human herpes virus-8 staining (Fig. 6) [2, 8, 9].

## Differential diagnoses

The heterogeneous clinical, radiographic and laboratory findings of KHE raise an extensive differential diagnosis that includes IH, venous malformation, congenital hemangioma and Kaposiform lymphangiomatosis (KLA), among others. A definitive preoperative differential diagnosis between deep KHE and a malignant tumor (e.g., metastasis neuroblastoma and sarcoma) is also challenging in patients with spine involvement without KMP.

### Infantile hemangioma

One of the most important diseases to rule out in the differential diagnosis of KHE is IH. The appearance of IH is dictated by the depth, location, and stage of evolution. A defining feature of IH is its dramatic growth (between 5.5 and 7.5 weeks of age) and development into a disorganized mass of blood vessels [86]. Cutaneous IHs are usually diagnosed clinically and frequently without needing for imaging. Imaging examinations and other investigations may be required in special situations (e.g., paraglottic or hepatic IHs). Both KHE and IH will look hypervascular on ultrasound. MRI is helpful in differentiating the difficult cases. Throughout the development of IHs, ECs in IH lesions are positive for Glut-1, which is absent in KHE and other vascular tumors [87, 88].

### Congenital hemangiomas

**Fig. 7:**
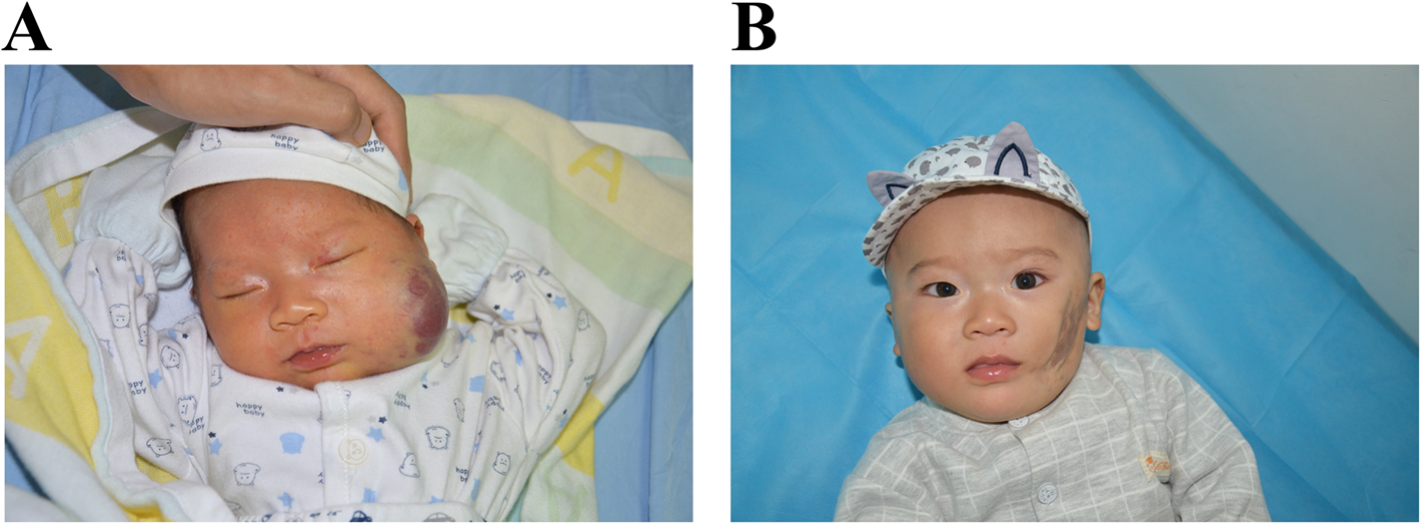
Rapidly involuting congenital hemangiomas (RICH). **a** RICH is fully formed at birth and then involutes, mostly during the first year of life. The patient’s hematologic parameters were within the normal reference ranges. **b** The same RICH involuted rapidly without any treatment. By 9 months of age, the lesion had involuted completely, leaving dermal atrophy

Congenital hemangiomas are biologically and behaviorally distinct from KHEs. They are benign vascular tumors of infancy that arise in utero and are present and fully formed at birth. The 3 variants of congenital hemangiomas are rapidly involuting congenital hemangiomas (RICHs), partially involuting congenital hemangiomas (PICHs) and noninvoluting congenital hemangiomas (NICHs) [89, 90]. The defining feature of RICHs is accelerated regression, which is usually complete within the first year of life, leaving behind atrophic skin (Fig. 7). NICHs persist in a stable state without growth or involution. However, a slight enlargement of NICHs over the years has recently been reported [91]. PICHs evolve from RICHs to persistent NICH-like lesions. Congenital hemangiomas can look very much like KHE particularly in the newborn period. Congenital hemangiomas do not exhibit progressive postnatal growth. In contrast, KHE tumors that develop KMP will appear to ‘grow’ and become engorged and purpuric in the first days/weeks/months of life. Mild consumption of coagulation factors can occur but most importantly, the coagulopathy associated with congenital hemangioma is not associated with bleeding issues and tends to self resolve in 1 to 2 weeks [92]. Treatment with surgical excision, as there are no medical therapies currently, for congenital hemangiomas may be required for cosmetic reasons or to resolve persistent pain in patients with NICH [89].

### Venous malformation

KHE with KMP should be differentiated from the clotting disorder associated with extensive venous malformations (VMs). VMs are slow-flow vascular malformations present at birth. In patients with extensive VMs, especially involving the trunk and/or extremities, localized intravascular coagulopathy can occur at baseline and be worsening by any aggravation of the malformation such as trauma or surgery. The levels of fibrinogen are low, associated with elevated D-dimer and fibrin degradation products. However, the thrombocytopenia is less profound in VMs than in KHE with KMP [93]. VMs can usually be diagnosed based on patient history, physical examination and MRI. On light microscopy, VMs are characterized by enlarged and irregular venous channels lined by a flattened layer of ECs [94].

### Kaposiform lymphangiomatosis

KLA is a rare, complex lymphatic disorder with multifocal or diffuse lesions. KLAs often involve the mediastinum, lung, abdomen and multiple bones [95–97]. Interestingly, thrombocytopenia and coagulopathy in KLA have overlapping features with KMP in KHE. The thrombocytopenia in some KLAs is extremely severe, similar to that observed in KMP [98, 99]. Histologically, KLAs are composed of malformed lymphatic channels with dispersed and poorly marginated clusters or sheets of spindled lymphatic ECs [96, 98]. In vitro, KHE cells can support vascular network formation, whereas KLA cells appear inert in this capacity [35]. A somatic activating *NRAS* variant (c.182 A > G, p. Q61R) was recently identified in patients with KLA but was absent in KHE samples, thus providing a molecular means to further differentiate these two entities [100]. In addition, KLA appears to be more refractory to medical therapies, with an overall survival rate of only 34% [96].

## Management

Due to the marked heterogeneity and rarity of KHEs, no validated scores have been established to assess disease severity. Consensus treatment statements by Drolet et al. [83] were published in 2013. Medical treatments with corticosteroids and/or vincristine have been recommended for the management of KHE. However, these recommendations are based on expert opinion rather than rigorous clinical studies. There is a lack of well-designed clinical trials and insufficient evidence to support existing interventions. There is also no definite treatment guideline for the long-term observation of patients with KHE [101].

Currently, the management of KHE has been based upon a review of the available evidence, expert opinions, and clinical experiences. Sirolimus has recently been suggested as a treatment option for complicated vascular anomalies and tumors in children, including KHE with or without KMP [102]. In many patients, multiple treatments are given in sequence or in combination. Notably, the treatment practices and regimens for KHE should be tailored to individual patients and guided by specific clinical circumstances. Patients with KMP should be treated aggressively with a combined regimen; monotherapy is usually not recommended. Several studies for combined regimens in the treatment of KHE are currently accruing subjects, including one randomized controlled trial (ClinicalTrials.gov identifier NCT03188068). Supportive care treatments (e.g., cryoprecipitate) are often required for patients with KMP (Table 1). Platelet transfusion should not be used unless the patient is actively bleeding or in preparation for surgery. Ideally, a patient with KHE who also exhibits severe complications or is at risk of complications should be referred to a multidisciplinary team for evaluation and treatment.

**Table 1:** Management options for KHE^a^

Specific approach	Dosage	Comment
Systemic		
Corticosteroids	Oral prednisolone 2 mg/kg/d or intravenous methylprednisolone 1.6 mg/kg/d	Corticosteroid plus vincristine or corticosteroid plus sirolimus is recommended as the first-line therapy for severe KMP. Corticosteroids should be tapered as soon as medically feasible.
Vincristine	Intravenous 0.05 mg/kg once weekly	Vincristine plus aspirin and/or ticlopidine and vincristine plus corticosteroid are recommended for severe KMP.
Sirolimus	Oral 0.8 mg/m^2^/dose twice daily, adjust for a trough level of 8–15 mg/ml	Sirolimus plus corticosteroid is now recommended as the first-line therapy for severe KMP. Lower serum levels (2–5 mg/ml) of sirolimus are recommended for long-term use or toxicities.
Ticlopidine	Oral 10 mg/kg/d	Monotherapy is not recommended for KMP.
Interferon-α	Subcutaneous injection, 1–3 × 10^6^ U/m^2^	Interferon-α is not recommended for patients younger than 1 year old due to its significant neurologic toxicities.
Propranolol	Oral 2–3 mg/kg/d	Monotherapy is not recommended for KMP.
Topical		
Sirolimus gel	0.1%, twice daily	Used for superficial KHE.
Tacrolimus ointment	0.1%, twice daily	Used for superficial KHE.
Supportive care treatments		
Fresh frozen plasma or cryoprecipitate	The dosage used is based on the actual situation of the patient (e.g., the severity of hypofibrinogenemia).	Used for active bleeding, platelet count < 30 × 10^9^/L, and/or fibrinogen < 1.0 g/L.
Platelets	The dosage used is based on the actual situation of the patient (e.g., the severity of thrombocytopenia).	Platelet transfusion is only recommended for active bleeding or before a surgical procedure when the platelet count is less than 30 × 10^9^/L.
Packed red blood cells	The dosage used is based on the actual situation of the patient (e.g., the severity of anemia).	Transfusion of packed red blood cells is recommended for patients who have symptomatic anemia (hemoglobin concentration less than 80 g/L).
Active nonintervention	Adjust scheduled visits on the basis of growth of the tumor and/or the development of complications.	Careful observation is recommended for unchanged and uncomplicated KHE.

^a^*KHE* Kaposiform hemangioendothelioma, *KMP* Kasabach-Merritt phenomenon

### Pharmacological treatments

#### Vincristine

Haisley-Royster et al. [103] reported encouraging findings regarding the successful use of vincristine in the management of KMP. Many studies have also demonstrated the satisfactory outcomes of vincristine in treating KHE with KMP, including steroid-resistant patients [5, 104–106]. First-line therapy with vincristine or vincristine plus corticosteroids is recommended for cases of KHE with KMP in consensus-derived guidelines [83, 107]. First-line treatment with vincristine has an overall response rate of 72% [108]. There is mounting evidence that vincristine monotherapy is not effective in very severe patients [109, 110]. In this regard, successful use of vincristine plus ticlopidine has been reported in some cases [5, 111].

#### Corticosteroids

Systemic corticosteroid treatment is recommended as another first-line therapy for KMP because of its success in rapidly normalizing platelet counts [83, 107]. The sustained response, however, is variable, and many cases do not improve with corticosteroid monotherapy (with an overall response rate of 10–27%), even when higher doses are given [106, 112]. In addition, long-term corticosteroid use has undesired side effects, such as temporary growth retardation, an increased risk of infection and behavioral changes [113]. Recent studies have suggested that corticosteroids can be used in combination therapies for KMP [73, 114]. Considering the undesirable side effects of long-term daily corticosteroid treatment in children, they should be weaned off these drugs as soon as medically feasible.

#### Sirolimus

**Fig. 8:**
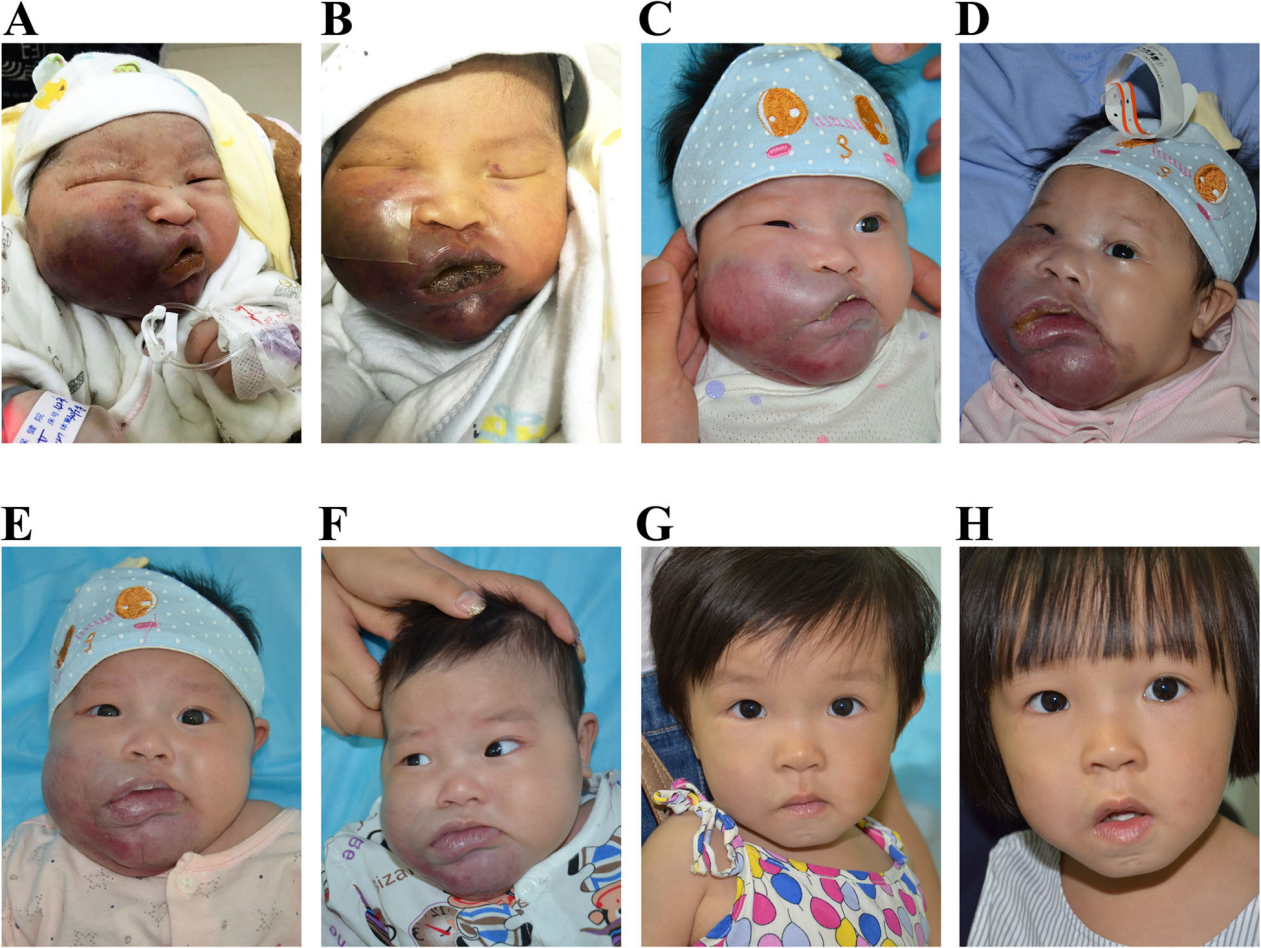
Congenital KHE associated with KMP on the right face. Congenital KHEs with KMP likely represent a period of temporary and partial remission shortly after birth. The signs and symptoms may alleviate spontaneously. However, rebound growth of the lesions accompanying severe KMP would reoccur within the next several days or weeks. **a** The parents’ photograph revealed a bluish, swollen and firm vascular mass on the right face after birth (2 days of age). Her platelet count was 7 × 10^9^/L. Without any special treatment, the tumor became soft and was stagnant in size. Her platelet count reached a highest value of 161 × 10^9^/L (**b**: 1 week of age, **c**: 6.5 weeks of age). Subsequently, however, the tumor became progressively enlarged and displayed obvious ecchymosis (**d**: 8 weeks of age). The patient’s platelet count dropped to 3 × 10^9^/L. She was treated with a combination therapy of sirolimus (0.8 mg/m^2^ administered twice daily) and prednisolone (2 mg/kg/d). One (**e**) and 4 weeks (**f**) after treatment. Within 10 days of combination therapy, the girl’s platelet level normalized. The prednisolone was tapered and discontinued within the following 4 weeks, and sirolimus was continued. G, Twelve months after treatment. H, Photograph at 26 months of age (24 months of treatment) demonstrates a nearly complete involution of the lesion. Sirolimus was then tapered and discontinued

Since 2010, an increasing number of studies have reported the exceptional effectiveness of sirolimus and everolimus, which are well-known mTOR inhibitors, on KHE. The authors described a reduction in KHE size, an eventual normalization of platelet counts in KMP patients, and in some cases, improvements in musculoskeletal pain, function and quality of life [80, 109, 112, 114–124]. For patients who either did not respond to the previous treatment (e.g., corticosteroids and vincristine) or who relapsed once the dose was tapered, sirolimus therapy still exhibited a high response rate (94%) [108]. However, sirolimus alone is usually not sufficient to treat severe KMP. In patients with severe KMP, sirolimus in combination with the short-term administration of corticosteroid has been recommended (Fig. 8) [114]. Sirolimus plus steroids is now considered as a first-line therapy for the treatment of KHE with KMP (versus vincristine plus steroids). Many clinicians (and families) prefer sirolimus (plus steroids) over vincristine because vincristine requires a central line.

Although sirolimus is clearly efficacious, rare side effects, such as interstitial pneumonitis and *Pneumocystis carinii* pneumonia, may be life-threatening [125, 126]. Currently, the optimal sirolimus dose and prophylactic regimen in patients with KHE has not been established. Many authors have reported maintaining the serum levels between 8 and 15 ng/ml [114, 116, 120]. However, low-dose sirolimus (2–3 ng/ml serum levels) may be associated with low toxicity and has been demonstrated to be effective for treating patients without KMP [127]. Interestingly, there is mounting evidence that low-dose sirolimus markedly ameliorates the development of inflammation and fibrogenesis in animal models, providing a theoretical basis for its use in KHE with musculoskeletal disorders [128, 129].

#### Topical treatments

Several case reports and case series have reported success using topical sirolimus and tacrolimus ointment in superficial KHE/TA. The investigators have shown a good response of KHE/TA to twice daily topical application of these drugs [130, 131]. Tacrolimus is an anti-T-cell immunosuppressive drug that is FDA-approved as a topical gel formulation (available in a concentration of 0.03 and 0.1%) for the treatment of cutaneous inflammatory/fibrotic diseases [132]. The clinically important implication of topical treatments is that superficial KHE/TA can be treated with local/topical agents, thus decreasing the potential complications associated with systemic treatments. However, it is important to note that most of these cases are TA lesions rather than KHE. It is also important to ensure that there is not a deep component.

#### Other pharmacological therapies

Several other medicines have been used in an attempt to optimize efficacy. Ticlopidine and aspirin are specific ant-platelet-aggregating agents. Successful use of ticlopidine plus aspirin in KMP has been described [133]. Interferon-α and propranolol have also been used to treat KHE. However, the standard protocols are inadequate because responses to these agents are variable and unpredictable [134–137]. In addition, the side effects of interferon-α are significant and include spastic diplegia [138].

### Invasive interventions

Elective resection of a KHE during the active phase of KMP is unusually not necessary and is ill advised. Given the young age of the patients and the vascularity of the tumors, these patients are at higher risk of blood loss and iatrogenic injury, along with worsening of the coagulopathy. Clinically, surgery is rarely an option for extensive KHEs or for patients in whom surgery will result in significant functional impairment. Conversely, surgery can be an approach for tumors in which a complete and safe resection can be performed [48]. Surgery is also an option for resection of a fibrofatty residuum or reconstruction of damaged structures [12]. Failure of pharmacotherapy may lower the threshold for the resection of a cosmetically or functionally problematic KHE [139]. In patients with KMP, arterial embolization may have a role in disease control [139]. Embolization can initially decrease blood flow from the tumor and decrease risk of high output cardiac failure. However, a remarkable limitation of embolization is the technical difficulty of cannulating very small feeder vessels in young patients. The possibility of worsening the hematological parameters by invasive interventions is also important and highlights the need for employing more established techniques in these patients.

## Conclusions and future directions

Although the incidence of KHE is low, it can cause morbidity and mortality in children and adults. Consequently, prompt diagnosis and appropriate management are crucial to improving the long-term prognosis of patients. Mutations and their pathways are providing potential targets for the development of novel pharmacotherapy for KHE. The future challenge will be to dissect the mutations and signaling cascades in terms of their pharmacological relevance. It is likely that the rapid advances in basic science and translational medicine will facilitate the development of important, new, and targeted molecular treatment strategies for KHE. Further clinical studies are also needed to refine the guidelines for the standard use of therapies and follow-up in patients with KHE.

## Data Availability

The datasets used and/or analyzed during the current study available from the corresponding author on reasonable request.

## Abbreviations

Ang-2: Angiopoietin-2
CLEC-2: C-type lectin-like receptor-2
CT: Computed tomography
GP: Glycoprotein
GPCR: G-protein-coupled receptor
IH: Infantile hemngioma
KHE: Kaposiform hemangioendothelioma
KLA: Kaposiform lymphangiomatosis
KMP: Kasabach-Merritt phenomenon
KMS: Kasabach-Merritt syndrome
MRI: Magnetic resonance imaging
MSC: Mesenchymal stem cell
NRP-2: Neuropilin-2
Prox-1: Prospero-related homeobox-1
TA: Tufted angioma
VEGF-C: Vascular endothelial growth factor-C
VEGFR-3: Vascular endothelial growth factor receptor-3
VM: Venous malformations
